# Removal of Tetracycline from Aqueous Solution Using Nanocomposite Based on Polyanion-Modified Laterite Material

**DOI:** 10.1155/2020/6623511

**Published:** 2020-12-11

**Authors:** Thi Hau Vu, Thi Mai Viet Ngo, Thi Tu Anh Duong, Thi Hien Lan Nguyen, Xuan Truong Mai, Thi Hong Nguyet Pham, Thi Phuong Le, Thi Hue Tran

**Affiliations:** Thai Nguyen University of Education, Thai Nguyen University, 20 Luong Ngoc Quyen, Quang Trung, Thai Nguyen, Vietnam

## Abstract

This work investigated the removal of antibiotic tetracycline (TC) from wastewater using nanocomposite material based on laterite modified with polyanion, polystyrene sulfonate (PSS). The effective factors influenced on the TC removal using nanocomposite PSS-modified laterite (NCPML) were optimized and found to be pH 4, solid-liquid ratio 5 mg/mL, and contact time 180 min. The highest removal of TC reached about 88% under the optimum adsorption conditions. The adsorption isotherm and kinetics of TC adsorption onto NCPML were in good agreement with the Langmuir and pseudo-second-order models, respectively. The characteristics of the NCPML material before and after TC adsorption were examined by zeta (*ζ*) potential measurements, Brunauer–Emmett–Teller (BET) method, and Fourier transform infrared spectroscopy (FT-IR). The TC adsorption onto NCPML was induced by electrostatic interaction, hydrogen bonding, and diffusion interaction. The TC removal from wastewater was approximately 94% while efficiency still reached 66% after five regenerations. Our research reveals that NCPML is a high-performance adsorbent for TC removal from wastewater.

## 1. Introduction

Nanocomposite material based on polymer-coated substrate is a hybrid adsorbent for pollutants removal [1–3]. Basically, the polycation adsorption onto negatively charged clays is forming the composite that is widely used in environmental remediation [4, 5]. Various organic contaminants such as herbicides [6], organic dyes [7], heavy metals [8], and antibiotics [9] were removed using these kinds of composite. On the contrary, polyanion adsorption onto positively charged minerals as a new composite material for antibiotics removal has not been reported.

**Figure 1:**
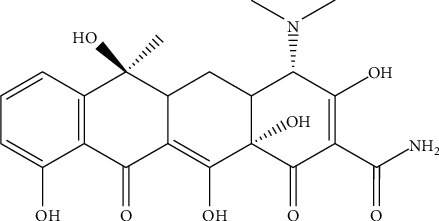
Molecular structure of TC.

In recent years, aquaculture in Vietnam has progressed on a large scale and production. However, aquaculture and fish processing have produced pollutants to the environment, especially to water sources. The wastewater from aquaculture contains decomposing surplus food sources, chemicals, and antibiotics. Many families of antibiotics are used for bacteria treatment [10]. Tetracycline (TC) is a big antibiotic group that is widely used to treat animal and human diseases as well as an antibacterial in aquaculture activity [11, 12]. In TC group, tetracycline (TC), whose molecular structure is shown in Figure 1, is the most common antibiotic. The TC is widely used to not only treat diseases of animals but also used as a part of animal feed to enhance effective growth [13]. High amounts of TC are released into the aqueous solution that can cause serious problems for animals and human health [14, 15]. Thus, various techniques are investigated to eliminate TC [16].

The well-known methods for TC removal include adsorption [13, 17–19], advanced oxidation [20], and degradation using catalyst [21] and membrane bioreactor [22–24]. It is evident that adsorption is a popular method used in developing countries because many minerals or natural soils can be fabricated or modified slow-cost adsorbents [17, 25–27]. Adsorption is known as a green technique in which fewer chemicals are used [28]. In addition, adsorption is a versatile method due to easy modification and regulation of charged surface. On the contrary, low selectivity with common adsorbent is a disadvantage of the adsorption technique [29]. Therefore, a new adsorbent for pollutants removal is always a great topic of interest for many scientists. To the best of our knowledge, the removal of TC by nanocomposite-based polyanion-coated laterite soil has not been investigated [16, 30].

Laterite is common in tropical countries like Vietnam. The material was modified by an anionic surfactant sodium dodecyl sulfate (SDS) for removal of different pollutants [31–33]. However, using polyanion such as polystyrene sulfonate (PSS) to modify laterite soil for TC removal is still a new study.

The interfacial and physicochemical methods including *ζ* potential measurements, Brunauer–Emmett–Teller (BET), and Fourier transform infrared spectroscopy (FT-IR) were employed to evaluate the changes in material characterization before and after TC adsorption. The isotherms and kinetics of TC onto NCPML were systematically investigated by experimental consideration and theoretical models. The reuse of NCPML and the application for TC removal in actual wastewater were also investigated in this work.

## 2. Materials and Methods

### 2.1. Materials

We collected the raw laterite at Thach That district, Hanoi, Vietnam. The bare laterite was treated as in our previously published papers [32, 33].

A strong polyelectrolyte with a negative charge, polystyrene sulfonate (PSS) with purity ≥ 98%, was purchased from Scharlau (Spain, EU). Tetracycline (TC) with purity ≥ 95% was supplied from Sigma-Aldrich (USA). We used monovalent salt of NaCl (p.A, Merck) to study the influence of ionic strength on the adsorption. In addition, strong acid HCl and strong base NaOH (p.A, Merck) were used to regulate the pH of all solutions. A pH meter (Presisa 900-Switzerland) using a commercial glass electrode was used to monitor pH for solutions. The deionized water was used and prepared for the aqueous solutions in this investigation.

### 2.2. Nanocomposite Material Based on PSS-Modified Laterite

Nanocomposite material was fabricated on the basis of PSS-modified laterite under the optimum condition. Firstly, the laterite was thoroughly mixed by shaking with 100 mg/L of PSS in 50 mM NaCl and the solid-liquid ratio 5 mg/mL at pH 4 for 150 min. Then, the solid material was separated by centrifuging before cleaning it with deionized water. The obtained nanocomposite-based PSS-modified laterite was formed and called as NCPML.

### 2.3. Adsorption Studies

Adsorption by the batch technique was used to study TC removal using NCPML. First of all, the stock solutions of TC with a concentration of 100 mg/L were prepared by dissolving the accuracy of TC amount in methanol and deionized water. Then, the stock solutions were taken for dilution to give a daily solution.

Different amounts of adsorbent were mixed with 25 mL TC solution of given concentrations in 100 mL Erlenmeyer flasks at 25 ± 2°C. Some important factors affecting the TC removal such as contact time, adsorbent dosages, pH of solution, and ionic strength were systematically investigated. All concentrations of TC in aqueous solutions were examined by the ultraviolet visible (UV-Vis) method at 277.4 nm using a UV-1700 spectrometer (Shimadzu, Japan). The TC removal efficiency was calculated by the following equation:

(1) $$\begin{array}{c} \text{removal efficiency }\left(\%\right)=\frac{C_{i}-C_{f}}{C_{i}}\times 100\%, \end{array}$$

where *C*_*i*_ and *C*_*f*_ are the initial and final concentrations of TC (mg/L).

The following equation was used to determine the TC adsorption capacity onto NCPML material:

(2) $$\begin{array}{c} \Gamma =\frac{C_{i}-\;C_{e}}{m}\times V, \end{array}$$

where Γ is denoted as adsorption capacity of TC (mg/g), *C*_*e*_ is the TC equilibrium concentration (mg/L), *V* is volume of solution (L), and *m* is the adsorbent mass (g).

### 2.4. Characterization Methods

The *ζ* potential measurements and Brunauer–Emmett–Teller (BET) method were employed to characterize NCPML material before and after TC adsorption.

Zeta (*ζ*) potential was conducted using a Nano ZS Zetasizer (Malvern, UK). The *ζ* potential was calculated by the following equation [34]:

(3) $$\begin{array}{c} \zeta =\frac{u_{e}\eta }{\varepsilon_{rs}\varepsilon_{0}}, \end{array}$$

where *u*_*e*_ is the electrophoretic mobility (*μ*ms^−1^/V·cm^−1^), *η* is the dynamic viscosity of the liquid (mPa·s), *ε*_0_ is the electric permittivity of vacuum (8.854 × 10^−12^·F/m), and *ε*_*rs*_ is the relative permittivity constant of the electrolyte solution.

The BET method is based on N_2_ adsorption and desorption isotherms on the material to determine the specific surface area of the NCPML before and after TC adsorption using a surface area analyzer, Micromeritics (TriStar 3000, Norcross, GA, USA).

Furthermore, we also used the Fourier transform infrared (FT-IR) spectroscopy to check the different vibration groups of the NCPML before and after TC adsorption. The FT-IR spectra were conducted by using Affinity-1S (Shimadzu, Japan).

### 2.5. Adsorption Isotherm and Kinetic Modeling

#### 2.5.1. Adsorption Isotherm Modeling

It is necessary to apply a suitable model to deeply understand the mechanisms of the adsorption process. The Langmuir and Freundlich models were applied to fit TC isotherms NCPML.

The Langmuir isotherm equation is [35] as follows:

(4) $$\begin{array}{c} \frac{C_{e}}{q_{e}}=\frac{C_{e}}{q_{\max}}+\;\frac{1}{q_{\max}K_{L}}, \end{array}$$

where *K*_*L*_ (L/g) is the Langmuir constant, *q*_max_ (mg/g) is the maximum amount, and *q*_*e*_ (mg/g) is the equilibrium amount of TC.

The equation described by the Freundlich model [36] is given in the following equation:

(5) $$\begin{array}{c} \ln\;q_{e}=\ln\;K_{F}+\frac{1}{n}\ln\;C_{e}, \end{array}$$

where the Freundlich constant is *K*_*F*_ (mg^*n*−1^L^*n*^/g) and the intensity of the adsorption is 1/*n*.

#### 2.5.2. Adsorption Kinetic Modeling

The pseudo-first-order and pseudo-second-order models are used in the present study.

The kinetic model of pseudo-first-order is

(6) $$\begin{array}{c} \log\left(q_{e}-q_{t}\right)=\log\;q_{e}-\frac{k_{1,k}}{2.303}t. \end{array}$$

The kinetic model of pseudo-second-order described by the following equation:

(7) $$\begin{array}{c} \frac{t}{q_{t}}=\frac{1}{k_{2,k}\cdot q_{e}^{2}}+\frac{1}{q_{e}}t, \end{array}$$

where *q*_*e*_ and *q*_*t*_ (mg/g) are adsorption capacities of TC onto NCPML at equilibrium and time *t*, respectively, and *k*_1,*k*_ (1/min) and *k*_2,*k*_ (g/mg·min) are reaction rate constants for pseudo-first-order and pseudo-second-order adsorption kinetics, respectively.

## 3. Results and Discussion

### 3.1. Effective Conditions for TC Adsorption onto NCPML

#### 3.1.1. Effect of pH

**Figure 2:**
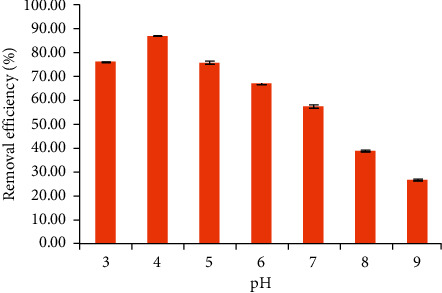
Effect of pH on the TC removal using NCPML (*C*_*i*_ (TC) = 10 mg/L). Error bars indicate the standard deviations of three replicates.

The effect of pH on the TC removal using NCPML is shown in Figure 2. The experiment of pH effect was conducted in the pH range of 3–9 to find out the best pH for TC removal using NCPML.

Figure 2 shows that the TC removal using NCPML reached the maximum at pH 4 and then decreased from pH 5 to 9. The p*K*_*a*_ values of TC are 3.3, 7.7, and 9.7; therefore, in the range of pH 3–9, TC exists in the cationic, zwitterionic, or anionic forms in aqueous solution under pH 3.3, in the range of pH 3.3–7.7, and above pH 7.7, respectively [37]. At pH 4, the negative charge surface of NCPML is convenient for attaching the cationic and zwitterionic species of TC. This result is similar to the previous report by other researchers [38]. When increasing the pH value from 5 to 8, the TC removal decreased because the negative part of the TC zwitterionic form increased. However, at pH ≥ 8, the decrease in adsorption was observed due to the increase in electrical repulsion force between the anionic TC and the negatively charged NCPML surface. At pH 3, a part of the metal oxide of laterite may be dissolved, so TC removal also decreased. The pH 4 is therefore selected and kept for TC removal using NCPML.

#### 3.1.2. Effect of Contact Time

**Figure 3:**
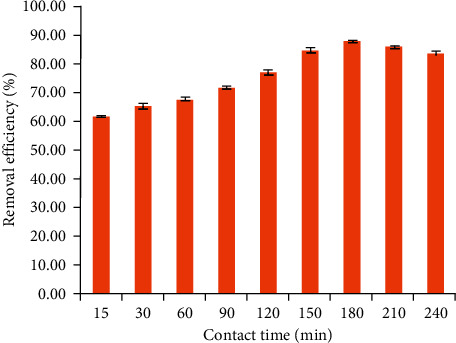
Effect of contact time to the TC removal onto NCPML (*C*_*i*_ (TC) = 10 mg/L), pH 4. Error bars indicate the standard deviations of three replicates.

The influence of contact time on TC removal using NCPML is shown in Figure 3. The contact time increased from 15 to 240 min. Figure 3 indicates that TC removal using NCPML grew up from 61 to 87% when increasing time in the prior 15–180 min. When contact time is greater than 180 min, the TC removal efficiency decreased insignificantly because of the PSS desorption from NCPML. In addition, the error bars of three replicates were the smallest at 180 min so that 180 min is the best contact time for TC removal.

#### 3.1.3. Influence of Adsorbent Dosage

**Figure 4:**
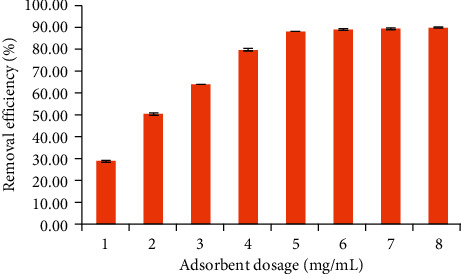
Effect of adsorbent dosage to the TC removal onto NCPML (*C*_*i*_ (TC) = 10 mg/L), pH 4, and contact time 180 min. Error bars indicate the standard deviations of three replicates.

It is necessary to study the influence of adsorbent dosage to evaluate the eco-effectiveness of adsorbents. The influence of adsorbent dosage on TC removal using NCPML was investigated in the range of 1–8 mg/mL and is indicated in Figure 4. As can be seen in Figure 4, the TC removal increased when the adsorbent dosage increased from 1 to 8 mg/mL as the result of rising the net surface charge or specific surface area of the adsorbent. The TC plateau removal efficiency was achieved at 5 mg/mL as 88% and was not changed significantly when the amount of adsorbent was above 5 mg/mL. Therefore, we used 5 mg/mL NCPML for further study on TC removal.

#### 3.1.4. Effect of Ionic Strength

**Figure 5:**
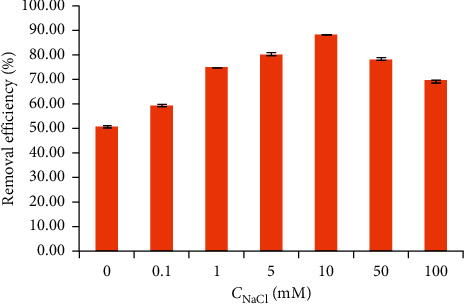
Effect of ionic strength to the TC removal onto NCPML (*C*_*i*_ (TC) = 10 mg/L), pH 4, contact time 180 min, and adsorbent dosage of 5 mg/mL. Error bars indicate the standard deviations of three replicates.

Ionic strength plays an important role in the Coulombic interaction between cationic, zwitterionic, or anionic forms of TC and the negative charge of NCPML.  Figure 5 shows that the TC removal efficiency increased when NaCl concentration increased from 0.1 to 10 mM and decreased from 10 to 100 mM NaCl. The results propose that TC removal is influenced by changing the concentration of NaCl. To deeply understand the influence of ionic strength on TC removal using NCPML, isothermal adsorption of TC onto NCPML is systematically investigated and given below.

### 3.2. Adsorption Isotherms and Mechanisms of TC onto NCPML

**Figure 6:**
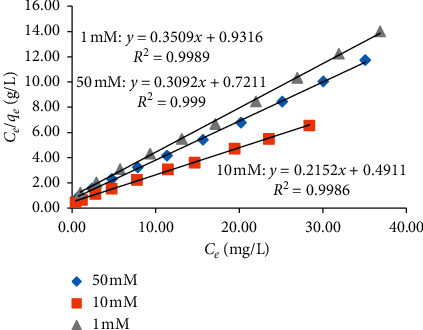
Adsorption isotherms of TC onto NCPML at three NaCl concentrations fitted by the Langmuir model.

**Figure 7:**
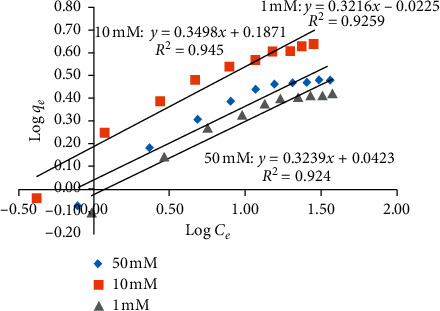
Adsorption isotherms of TC onto NCPML at three NaCl concentrations fitted by the Freundlich model.

Isothermal adsorption of TC onto NCPML at different ionic strengths in the presence of NaCl salt was an experimental study and fitted by Langmuir and Freundlich models (Figures 6 and 7). The main parameters of isothermal adsorption of TC onto NCPML at 25 ± 2°C are presented in Table 1. Table 1 and Figures 6 and 7 show that the isothermal adsorption of TC onto NCPML represented by the Langmuir model was better than the Freundlich one. The values of correlation coefficients when using Langmuir model (*R*^2^ > 0.99) were greater than that using the Freundlich one (*R*^2^ > 0.92).

Figures 6 and 7 show that the ionic strength strongly influenced the adsorption isotherms of TC onto NCPML. Adsorption increased with increasing NaCl concentration from 1 to 10 mM while this trend was reversed when increasing salt in the range of 10–50 mM. The higher the salt is, the lower the electrostatic attraction induced the adsorption. It suggests that TC adsorption onto NCPML was controlled by both electrostatic and nonelectrostatic interaction at low-ionic strength, but the electrostatic attraction was the main driving force for TC adsorption at high-ionic strength.

#### 3.2.1. Adsorption Mechanisms of TC onto NCPML

Adsorption mechanism of TC onto NCPML was predicted on the basis of FT-IR spectroscopy and the BET methods as well as *ζ* potential measurements.

**Figure 8:**
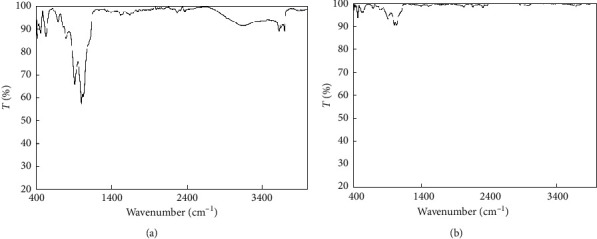
The FT-IR spectra of NCPML before (a) and after (b) TC adsorption in the wavenumber from 400 cm^−1^ to 4000 cm^−1^.

Figure 8 indicates that FT-IR spectra of NCPML and NCPML after TC adsorption are similar. Nevertheless, the alkyl chain assigned at 2927.94 cm^−1^ of NCPML did not appear at the FT-IR spectra of NCPML after TC adsorption. Moreover, many new peaks in the wave numbers from 1200 to 2200 cm^−1^ appeared in the spectra of NCPML after TC adsorption. The assigned peaks at 1747.51 cm^−1^, 1512.19 cm^−1^, and 1255.66 cm^−1^ occurred due to the appearance of C=O stretching, O-H deformation, and NH_2_ bending, respectively [39]. The FT-IR spectra show the occurrence of TC on NCPML surface via hydrophobic interactions between carbon chains of TC and natural organic matter of NCPML.

**Figure 9:**
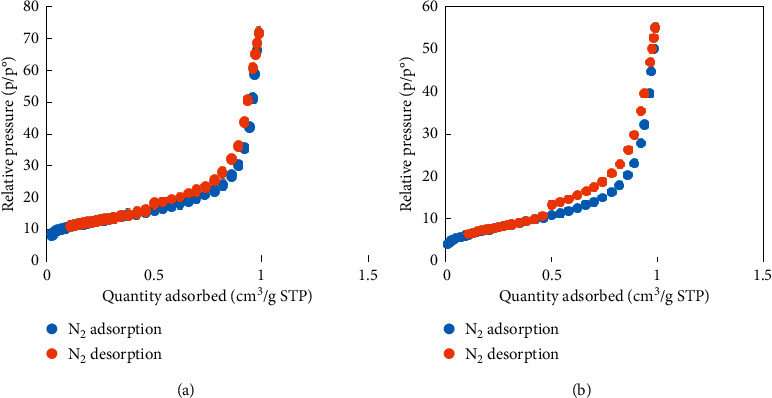
Nitrogen adsorption-desorption isotherms on NCPML before (a) and after (b) TC adsorption.

Figure 9 shows the BET results of NCPML before and after TC adsorption. As can be seen in Figure 9, the specific surface area of NCPML decreased about 15 m^2^/g after TC adsorption (a decrease from 42.39 m^2^/g to 27.51 m^2^/g). The average pore width of PSS-modified laterite which was examined by using the Barrett–Joyner–Halenda (BJH) model [40] to the N_2_ adsorption and desorption isotherms was 15.2 and 12.6 nm, respectively. These pores were easily determined by N_2_-BET (BJH) method demonstrated that our material is a nanocomposite. After TC adsorption, the average pore decreased and was found to be 10 nm. The BET results indicate that TC molecules were successfully taken up on NCPML.

**Figure 10:**
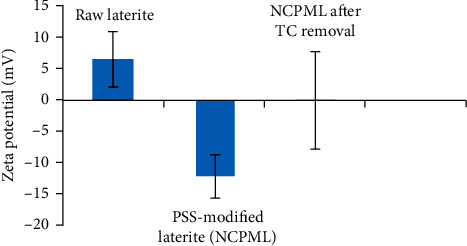
The *ζ* potential of laterite soil and PSS-modified laterite (NCPML) and NCPML after TC adsorption (pH 4).

The *ζ* potential is a powerful tool to evaluate the charging behavior of various materials through the adsorption technique by many researchers [41–44]. In our research, we also used the *ζ* potential for the purpose of confirmation of the TC adsorption mechanism onto NCPML. The *ζ* potential of laterite, laterite-modified PSS (NCPML), and laterite-modified PSS after TC adsorption was measured at pH 4.  Figure 10 shows that the *ζ* potential of raw laterite was positive (*ζ* = 6.49 mV), and the *ζ* potential of NCPML was negative due to the presence of PSS molecules (*ζ* = −12.0 mV). After TC adsorption, the NCPML was almost noncharged with the *ζ* potential of 0.121 mV. The *ζ* potential measurements results indicated that the adsorption is controlled by Coulombic interaction between positive and zwitterionic species of TC and negatively charged NCPML surface.

#### 3.2.2. Adsorption Kinetics of TC onto NCPML

In this section, we studied the adsorption kinetics of TC onto NCPML with different TC concentrations. The data for pseudo-first-order and pseudo-second-order models are presented in Table 2.

Table 2 shows that the pseudo-second-order is better than the pseudo-first-order, expressing by the higher correlation coefficients (*R*^2^) for pseudo-second-order at all TC concentrations. Moreover, the calculated *q*_*e*_ values by the pseudo-second-order (1.81, 3.56, and 5.25 mg/g for initial TC concentration of 10, 25, and 50 mg/L, respectively) are similar to experimental ones (1.76, 3.46, and 5.11 mg/g for 10, 25, and 50 mg/L, respectively). It implies that kinetic adsorption of TC onto NCPML agreed well with the pseudo-second-order model.

### 3.3. Adsorptive Removal of TC onto Laterite with and without Modification by PSS

**Figure 11:**
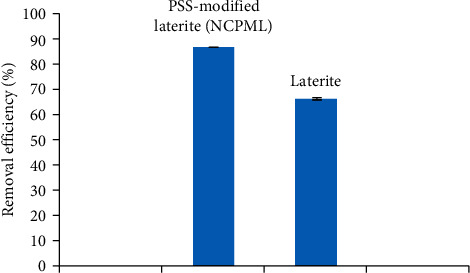
Removal efficiency of TC onto laterite with and without modification by PSS. Error bars show standard deviation of three replicates.

Removal efficiency of TC onto laterite with and without modification by PSS is presented in Figure 11. Figure 11 shows that the removal of TC, in which TC initial concentration is 10 mg/L in 10 mM NaCl and pH 4 increases from 66.67% to 87.09% after surface modification of laterite by PSS. The negative charge of laterite after surface modification enhanced the TC removal efficiency through electrostatic attraction between the positive and zwitterionic species of TC and the negatively charged NCPML surface. The result denotes that laterite-modified PSS is more effective than laterite soil in TC removal.

### 3.4. Reuse Potential of NCPML

**Figure 12:**
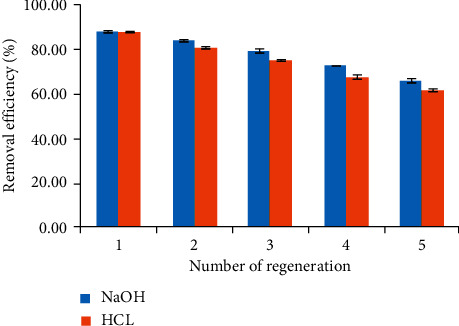
The removal of TC using NCPML after five regenerations. Error bars show standard deviation of three replicates.

It is necessary to investigate the regeneration of materials to evaluate the stability and reusable potential. For the material recovery procedure, we conducted the experiment by the following steps. First of all, the NCPML was desorbed by 0.1 M NaOH and 0.1 M HCl after TC adsorption at a concentration of 10 mg/L. Then, we used a centrifugal machine to separate the NCPML and liquid. After that, the NCPML was washed with ultrapure water many times to reach the pH neutral. In the next step, the NCPML was reactivated again with PSS residual (Section 2.2.). Finally, the NCPML was used again to remove 10 mg/L TC under the best conditions (Section 3.1.). The reuse of NCPML material by using HCl and NaOH was conducted at the same conditions and was repeated five times.  Figure 12 shows the TC removal using NCPML after five regenerations. It is clear to observe that TC removal was decreased with the number of recycles, but the removal still reached 61% and 66% after five regenerations for HCl and NaOH, respectively. Our results demonstrate that NCPML is not only low-cost but also reusable material.

### 3.5. Removal of Tetracycline from Wastewater

**Figure 13:**
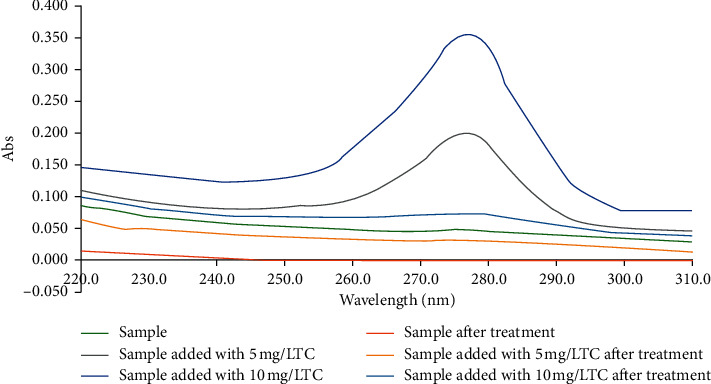
The UV-Vis spectrum of TC antibiotic in a water sample collected from aquaculture pond before and after treatment using NCPML.

In this study, we also applied NCPML to treat TC in an actual water sample. The water sample was collected from a fish aquaculture pond in Thai Nguyen Province. The sample was preserved in a polyethylene bottle in a freezer, and then, it was conveyed to the laboratory. We conducted the TC removal in the sample within 2 days. The TC removal from the sample was carried out at pH 4 and contact time 180 min. The UV-Vis spectrum of TC antibiotic in the sample before and after treatment as well as before and after adding TC standards using NCPML is shown in Figure 13. The TC absorbance at a wavelength of 277.4 nm deeply decreases because of the influence of various factors in actual water during the adsorption process.. By calculation, the TC removal efficiencies reached 81%, 78%, and approximately 94% for sample added with 5.0 mg/L TC standard; sample added with 10.0 mg/L TC standard, and only sample, respectively. The high removal efficiency of approximately 94% in treating TC in a fish aquaculture pond was obtained. The result indicated that NCPML is eco-effective for treating TC antibiotics from the water environment because of simple modification and high-removal efficiency.

## 4. Conclusions

This study investigated tetracycline removal from aqueous solution using nanocomposite-based PSS-modified laterite (NCPML). Different physicochemical and interfacial methods including *ζ* potential, FT-IR, and BET were used to evaluate adsorptive removal of TC using NCPML. The selected conditions for TC removal were found to be pH 4, adsorbent dosage 5 mg/mL, contact time 180 min, and 10 mM NaCl. Adsorption mechanisms of TC onto NCPML were controlled by both electrostatic attractions between negatively charged NCPML surface and the zwitterionic species of TC and nonelectrostatic interactions. Adsorption isotherms of TC onto NCPML were in accordance with the Langmuir model while adsorption kinetics agreed well with the pseudo-second-order model. The TC removal using NCPML was obtained above 66% after five recycles. A high-removal efficiency of TC in a real water sample collected from a fish aquaculture pond using NCPML reached approximately 94%. This work reveals that NCPML is a novel and hybrid adsorbent for the removal of antibiotics from the water environment.

## Figures and Tables

**Table 1:** Parameters of TC adsorption isotherms onto NCPML at different NaCl concentrations using Langmuir and Freundlich models.

Isothermal model	Parameters	NaCl concentration (mM)		
		1	10	50
Langmuir	*q*_max_ (mg/g)	2.85	4.65	3.23
	*K*_*L*_ (L/g)	0.38	0.44	0.43
	*R* ^2^	0.998	0.998	0.999

Freundlich	*K*_*F*_	0.95	1.54	1.10
	1/*n*	0.321	0.349	0.323
	*R* ^2^	0.925	0.945	0.924

**Table 2:** Parameters of adsorption kinetics of TC onto NCPML.

Kinetic model	Parameters	TC concentration (mg/L)		
		10	25	50
Pseudo-first-order	*q*_*e*_ (mg/g)	0.66	1.28	1.81
	*k*_1,*k*_ (1/min)	0.001	0.001	0.001
	*R* ^2^	0.826	0.830	0.845

Pseudo-second-order	*q*_*e*_ (mg/g)	1.81	3.56	5.25
	*k*_2,*k*_ (g/mg·min)	0.038	0.020	0.015
	*R* ^2^	0.992	0.995	0.996

## Data Availability

All the data and supporting materials used in this study are included within the article.
